# The Effect of Exogenous Surfactant on Moderate and Severe Stages of COVID-19 Induced ARDS: the Pilot Study of a Clinical Trial

**DOI:** 10.22037/ijpr.2021.115390.15347

**Published:** 2021

**Authors:** Kamal Fani, Mehdi Ghahremani, Mohammad Fathi, Nilofar Massoudi, Sasan Tavana, Navid Nooraee, Nasser Malekpour Alamdari, Sara Besharat, Arash Najafi Abrandabadi, Ali Pirsalehi, Mohammad Ali Khabiri Khatiri, Maryam Amini Pouya, Samira Rajaei, Ali Dabbagh

**Affiliations:** a *Anesthesiology Research Center, School of Medicine, Shahid Beheshti University of Medical Sciences, Tehran, Iran. *; b *Department of Internal Medicine, School of Medicine, Shahid Beheshti University of Medical Sciences, Tehran, Iran. *; c *Department of Surgery, School of Medicine, Shahid Beheshti University of Medical Sciences, Tehran, Iran. *; d *Department of Radiology, School of Medicine, Shahid Beheshti University of Medical Sciences, Tehran, Iran. *; e *Department of Pharmaceutics, Faculty of Pharmacy, Tehran University of Medical Sciences, Tehran, Iran. *; f *Department of Immunology, School of Medicine, Tehran University of Medical Sciences, Tehran, Iran.*

**Keywords:** COVID-19, Surfactant, Pulmonary involvement, ARDS

## Abstract

COVID-19 pandemic has created a global health challenge. Many pharmaceuticals have been repurposed as potential treatments, though many have not been promising. Due to the inflammatory and destructive effects of the virus on alveolar cells, the effect of exogenous surfactant was assessed as a potential treatment of lung dysfunction in COVID-19 patients. In this pilot study of the clinical trial, 49 patients aged 35-80 years with COVID-19 admitted in ICU entered the study (22 patients intubated and 23 had face masks; 4 patients in the control arm). The treatment arm patients received two consecutive doses of surfactant. P/F ratio (based on serial blood gas analyses before and 12 hours after 2 doses of surfactant) and also, clinical outcomes were assessed.in COVID-19 adult patients, surfactant significantly improved pulmonary P/F ratio both in intubated and face mask COVID-19 patients (increasing from 119.2 ± 51.7 to 179.4 ± 115.5). The rate of extubation was much better than similar country-wide studies. Surfactant significantly alleviates the respiratory status in moderate to severe COVID-19 ARDS with two consecutive 100 mg doses of surfactant (with 6 hours’ interval) though previous studies have been controversial, regarding the effect of surfactant in general forms of ARDS. Higher doses might have better effects, mandating more trials.

## Introduction

COVID-19 outbreak due to the novel coronavirus SARS-CoV-2 started in China in late 2019, transforming very rapidly to a dramatic global pandemic with great health and societal impacts. Pulmonary involvement remains among its serious complications (1-3).

Many pharmacological and non-pharmacological agents have been proposed as treatment hopes; none have been conclusive yet, necessitating more research (4-6). Many of the current therapeutic options are repurposed potential pharmaceuticals (7-10). 

Meanwhile, “hyaline membrane formation lesions indicating acute respiratory distress syndrome (ARDS)” are among the postmortem pathologic findings in pulmonary samples of COVID-19 patients; these lesions “much resemble the lungs of wet drowning” (11, 12).

Surfactant, combined of proteins and lipids, secreted into the alveolar space by type II alveolar cells, reduces alveolar surface tension and modulates immunological homeostasis and inflammatory mediators in the alveoli; these mechanisms are failed in alveoli of COVID-19 patients, partially due to surfactant shortage after the destruction of alveolar cells by virus-induced lung injury, leading a floating state in alveoli rendering gas exchange and augmenting inflammatory cascade (12-15). 

This study was performed to assess the effects of intratracheal and inhalational surfactant on the pulmonary status and clinical outcome of moderate and severe stages of ARDS in COVID-19 patients.

## Experimental

*Methods*

Research Ethics Committee approved the study proposal, Vice-Chancellor of Research Affairs March 28, 2020, coded IR.SBMU.RETECH.REC.1399.016. Besides, the protocol was approved by the Iranian Clinical Registry (https://www.irct.ir/) “IRCT20091201002804N12” on 06/01/2020. Informed written consent was taken from all the patients or when unconscious, from their relatives. Patient recruitment started immediately after 06/01/2020 in COVID-19 patients to Intensive Care Units in two university hospitals in Tehran, Iran.

The primary protocol is published before (16). The recruitment of the patients started with a pre-pilot phase, in which the patients were randomly assigned into two groups; *i.e*. they received Surfactant or placebo using an intra-tracheal approach. However, after assessing the results of the first group of patients (4 patients in each group), due to the significant clinical improvements seen in patients who had received surfactant, the researchers decided not to deprive anyone of the benefits of the study drug (surfactant), shifting all the patients to the surfactant group which could be considered as compassionate treatment. So, the current manuscript includes a pre-pilot and the “treatment arm of the study” though the authors know this might decrease the external validity of the study. A summary of the designed patient flowchart and its changes are presented in the CONSORT flow chart of this manuscript.

The clinical diagnosis and treatment protocol, including imaging and laboratory diagnostic features and therapeutic approaches, strictly followed the National Iranian Guideline for Diagnosis and Treatment of Adult COVID-19 Patients (first released on February 29, 2019) and its updates throughout the study (17-21). The diagnosis was confirmed using a combination of clinical and nasopharyngeal swab sampling RT-PCR test, added with a list of para-clinical tests including imaging studies; besides, serial arterial blood gas assessment was performed to manage the respiratory status. CT scanning studies were not included in all the study patients; because not all of the patients tolerated being transferred to the CT unit; though in a large number of the patients, CT was used for clinical purposes; meanwhile, all of the patients had at least portable chest X rays.

*Inclusion criteria were*

Adult COVID-19 patients admitted to ICU (age range of 18 to 80 years), moderate to severe ARDS (based on the definition of P/F ratio), it was mandatory to use auxiliary respiratory devices (including either intubation or face mask), ARDS was due to COVID-19

*Exclusion criteria were*

Any patient needing extracorporeal membrane oxygenation (ECMO), ARDS was primarily due to any other reason rather than COVID-19, the primary source of pulmonary involvement was bacterial pneumonia or any other etiology except for COVID-10 induced lung involvement, those who refused to continue the study (either the patient or his/her family), any patient had any sign of healing before entering the study leading to patient discharge from ICU in less than 12 h.

All the patients received exogenous surfactant in 2 doses with 6 hours’ intervals; each dose included 100 mg surfactant in 4 mL vials. In those who were intubated, the drug was administered directly into the endotracheal tube, and in the others, a constant face mask with a nebulizer was used.

For outcome measurement, the results of the proportion of Arterial Pressure of Oxygen to Inspirational Fraction of Oxygen (*i.e*. PaO2/FiO2 or simply P/F ratio) were analyzed using arterial blood gas samples throughout the study. The first P/F ratio was checked before administering surfactant (called P/F ratio 1). In contrast, the second P/F ratio was checked 12 hours after administration of the 2^nd^ surfactant doses (called P/F ratio 2). A *P*-value of less than 0.05 was considered significant. A T-test was used to compare the results.

## Results

A total of 55 patients entered the study while 6 were excluded (5 did not meet the inclusion criteria and one declined to participate); however, 49 patients remained including 8 in the pre-pilot stage and 41 in the treatment arm. The current data represent the 45 patients who were related to the treatment arm (4+41) of the study. The results of the data analysis are presented in Table 1. 

There was a statistically significant difference in the trend of the P/F ratio after surfactant administration; in both intratracheal and inhalation modes. Besides, among the 22 intubated patients, 7 were extubated and transferred to the ward after their course improved; 15 others have died; the extubation discharge was much higher than the total extubation rates in other studies; both compared with results of another cohort in the same geographic and demographic setting and the results of studies country-wide (22-24). The data of the 7 extubated patients are summarized in Table 2. 

## Discussion

The results of our study demonstrated that a low dose regimen of surfactant administered either through intratracheal route or by inhalational method (nebulizing face mask) could improve respiratory function in COVID-19 induced ARDS. To the best of our knowledge, this is the first clinical study assessing the role of exogenous surfactants in COVID-19 attributed ARDS patients. Surfactant seems effective in alleviating the respiratory status in moderate to severe COVID-19 ARDS with two consecutive 100 mg doses of surfactant (with 6 hours’ interval), though previous studies have been controversial regarding the effect of surfactant in general forms of ARDS. However, among other designed clinical studies, none has been finished yet and this is the earliest registered one, approved on March 28, 2020, and patient recruitment started immediately afterward.

The results of this study demonstrated that surfactant could improve the P/F ratio of the patients, increase the chance of extubation in intubated patients and decrease the chance of intubation in non-intubated cases compared with a larger sample in the same geographic and demographic setting; in which 459 confirmed COVID-19 patients were assessed in which 396 survived and 63 expired (22). While the latter study was performed in Tehran, Iran, similar findings were demonstrated in similar studies from Tehran and Isfahan, two major cities in Iran (23, 24). Compared with studies in a similar level of care, geographic and demographic status, our results demonstrate a significant role for surfactant in COVID-19 pneumonia patients. 

In COVID-19 patients, Weaning from mechanical ventilation and extubation are among the main determinant of outcome (25, 26). Besides, we considered the P/F ratio as one of our main indicators due to its role in defining ARDS severity (26-28).

Before the COVID-19 pandemic, there was a great controversy regarding the role of surfactants in ARDS patients, especially in adult patients; many studies failed to demonstrate any benefit from surfactant administration in adult ARDS regarding mortality rate, ventilator-free days, or improvement in oxygenation status (29, 30), or even might improve the oxygenation status of adult ARDS but could not improve mortality (31, 32). 

However, the current study demonstrated that in COVID-19 patients with moderate and severe stages of ARDS, when we consider the pulmonary effects of exogenous surfactant, things go a bit differently. Part of the explanation might result from the differences in the mechanisms of COVID-19 and the resulting cytokine storm, which has specific features for COVID-19 lung involvement, much different from other versions of adult ARDS (33-36). Also, the pathologic findings in COVID-19 patients’ lungs, resemble the findings in water drowning alveoli, added with “hyaline membrane formation lesions,” are among the postmortem pathologic findings in pulmonary samples COVID-19 patients (11, 12). These findings might be among the main explanations for the results of exogenous surfactant effectiveness in COVID-19 patients with moderate and severe stages of ARDS. From a demographic point of view, our results confirmed improved outcomes for critical patients compared with similar studies in the same country where there was no use of exogenous surfactant use in COIVD-19 ARDS (22-24).

Finally, we used a very low dose of surfactant yet received acceptable results. Compared with neonates receiving surfactants with respiratory distress syndrome, this dose was much lower (37-39). However, there might be some explanations: in neonates, there is a total paucity of surfactant in neonatal lung tissue; while, this is not the same case in COVID-19 attributed ARDS patients (11, 12). Possibly, this could be the reason why our doses have been relatively effective. However, more experiments with higher doses of surfactant might be even more promising.

**Table 1:** Data analysis of the study patients

	**Intubated (received intra-tracheal surfactant)**	**Non-intubated (received surfactant through nebulizer)**	**Global study data**
Number of patients	22	23	45 (41+4)
Age in years (mean ± standard deviation)	58.1 ± 15.2	65.2 ± 14.8	61.6 ± 15
Minimum/Maximum of Age in years	38/80	34/80	35/80
Female/Male ratio	8/14	10/13	18/27
P/F ratio 1^*^	78.6 ± 44.1	159.7 ± 59.3	119.2 ± 51.7
P/F ratio 2^**^	143.2 ± 116.6	214.4 ± 105.7	179.4 ± 115.5
*P*-value (between P/F ratio 1 & P/F ratio 2)	0.000 (df = 21)	0.000 (df = 22)	0.000 (df = 44)

^* ^P/F ratio 1: PaO2/FiO2 *i.e.* Arterial Pressure of Oxygen to Inspirational Fraction of Oxygen just before starting surfactant. ^**^P/F ratio 2: PaO2/FiO2 *i.e.* Arterial Pressure of Oxygen to Inspirational Fraction of Oxygen 12 hours after surfactant administration

**Table 2:** Data of the extubated patients

**Patient No**	**Age (years)**	**Gender**	**Duration of intubation (Days)**	**Lowest saturation level ever**	**Days to extubation after surfactant administration**
1	80	Male	6	40	5
2	48	Male	3	30	2
3	50	Male	3	60	2
4	41	Male	4	52	3
5	50	Male	4	30	3
6	51	Female	6	35	3
7	39	Male	4	41	4

## Limitations of the study

There are some limitations to this study. 

For ethical reasons, we changed the design of the study from a clinical trial to a “before-after one”.Though we tried not to deprive any of our patients of exogenous surfactant after the primary stage of the study, we could not assess our results in a clinical trial setting. 

We did not assess the postmortem lung tissues of the patients who had been expired, albeit had received exogenous surfactant. Possibly this could help future studies in uncovering the role of surfactants. There are not enough studies concerning the role of surfactants on lung tissues in COVID-19 patients mainly due to the novelty of the disease (40); in such a way that before launching this study, no other clinical studies were discussing the issue; so, this study could be considered as “a compassionate therapy one “. We did not compare the Chest x-rays, which could be considered as one defect.

Finally, we have not followed our patients yet, considering their respiratory indices compared with other patients who could have important findings. However, we tried to report our results to possibly benefit more patients worldwide.

## Conflict of Interest

The surfactant vials were provided by Tekzima Drug Alborz Company, Tehran, Iran, which provided the drugs as support to the study. The drug was provided as “Beraksurf “ as 100 mg in 4 mL vials. Except for the drugs, there is no relationship between the researchers, the latter company, or any other company.

## Ethics approval and consent to participate

 The study proposal was approved by the Research Ethics Committee, Vice-Chancellor of Research Affairs March 28, 2020, coded IR.SBMU.RETECH.REC.1399.016. Besides, the protocol was approved by the Iranian Clinical Registry (https://www.irct.ir/) “IRCT20091201002804N12”. Informed written consent was taken from all the patients or when unconscious, from their relatives

## Availability of data and material

Ali Dabbagh holds all the data available; besides, the majority of the data are referenced in previous studies cited throughout the text

## Competing interests

 The surfactant vials were provided by Tekzima Drug Alborz Company, Tehran, Iran which provided the drugs as support to the study. The drug was provided as “Beraksurf “ as 100 mg in 4 mL vials. Except for the drugs, there is no relationship between the researchers and the latter company or any other company.

## Funding

This research project is funded by the Anesthesiology Research Center, SBMU, Tehran, Iran with grant number 22959. Besides, support was provided solely from institutional and/or departmental sources.
